# P-435. Utility of Second Sample Testing in Indian Children Suffering from Tuberculosis with Rifampicin Indeterminate Results on Xpert MTB/Rif and Xpert Ultra Assays

**DOI:** 10.1093/ofid/ofaf695.651

**Published:** 2026-01-11

**Authors:** Dhruv Gandhi, Viren Amesur, Vaidehi Mehta, Sonal Patil, Dhruv Mamtora, Ira Shah

**Affiliations:** Bai Jerbai Wadia Hospital for Children, Mumbai, India, West Monroe, LA; Bai Jerbai Wadia Hospital for Children, Mumbai, India, West Monroe, LA; Bai Jerbai Wadia Hospital for Children, Mumbai, India, West Monroe, LA; Bai Jerbai Wadia Hospital for Children, Mumbai, India, West Monroe, LA; Bai Jerbai Wadia Hospital for Children, Mumbai, India, West Monroe, LA; Bai Jerbai Wadia Hospital for Children, Mumbai, India, West Monroe, LA

## Abstract

**Background:**

Nucleic acid amplification tests (NAAT) for tuberculosis(TB) such as Xpert MTB/Rif and Xpert Ultra, can rapidly detect *Mycobacterium tuberculosis* (MTB) and rifampicin resistance (RR). However, they may yield rifampicin indeterminate (RI) results which pose a significant challenge. Current guidelines suggest performing NAAT on a second sample and treating with first-line antitubercular therapy (ATT) until actionable results are obtained on NAAT or other drug-resistance assays. The aim of this study is to determine the detection yield, rifampicin resistance results, and clinical implications of second sample testing on NAAT in patients with first sample RI results.

**Table 1:**
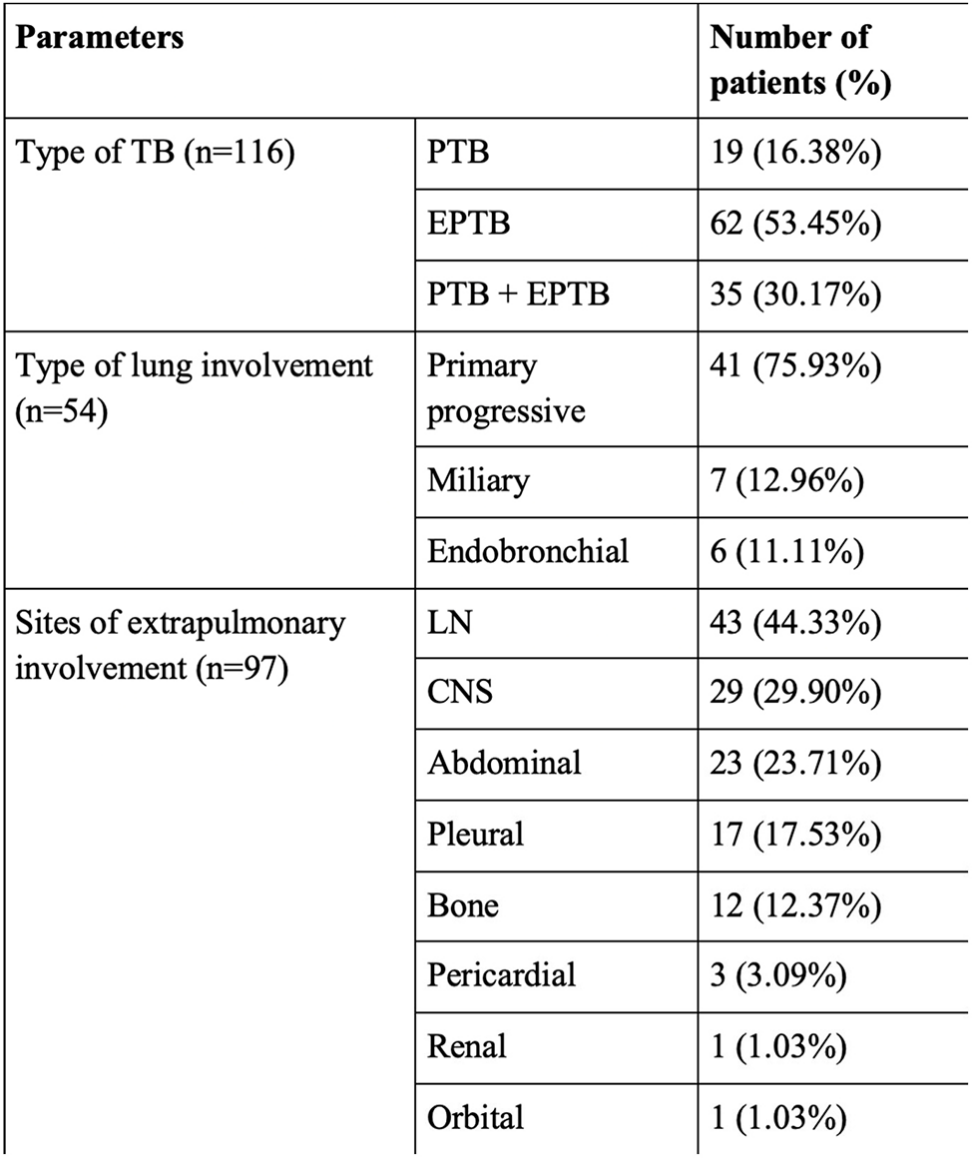
Clinical characteristics of the patients Note: TB: Tuberculosis, PTB: Pulmonary tuberculosis, EPTB: Extrapulmonary tuberculosis, LN: Lymph node, CNS: Central nervous system.

**Table 2:**
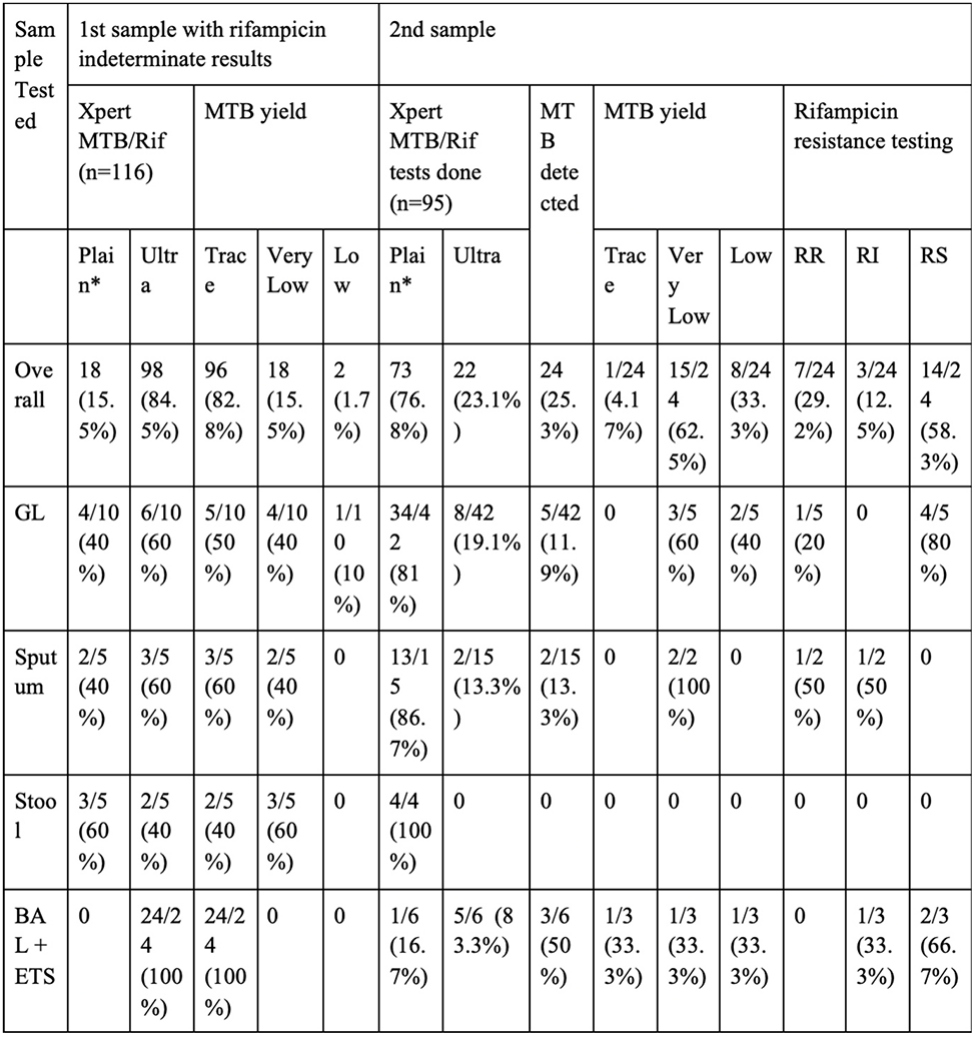
Xpert MTB/Rif and Ultra results of first and second samples (overall and pulmonary) Note: *Plain- refers to Xpert MTB/Rif assay, MTB- Mycobacterium tuberculosis, GL- Gastric lavage, BAL- Bronchoalveolar lavage, ETS- Endotracheal secretions.

**Methods:**

A retrospective study was conducted including all pediatric TB patients with RI results on their first sample NAAT. Data on specimen type, and second sample NAAT results was analysed.

**Table 3:**
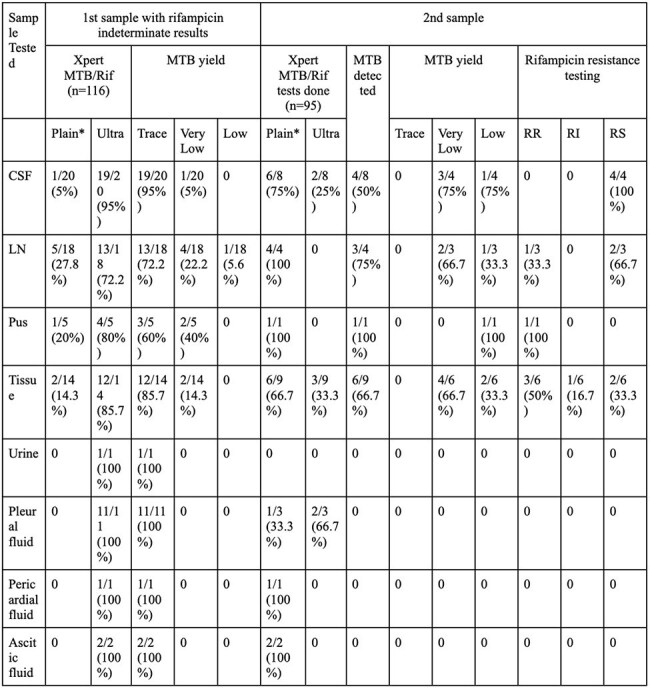
Xpert MTB/Rif and Ultra results of first and second samples (extrapulmonary) Note: *Plain- refers to Xpert MTB/Rif assay, MTB- Mycobacterium tuberculosis, CSF- Cerebrospinal fluid, LN- Lymph node.

**Results:**

One-hundred and sixteen patients were included. Second sample testing was done in 95 (81.90%) patients, of which 67 (70.5%) samples were pulmonary and 28 (29.5%) samples were extrapulmonary. For pulmonary samples, 10 (14.93%) detected MTB, of which 2 (20%) were RI, 6 (60%) were rifampicin sensitive (RS) and 2 (20%) were RR. For extrapulmonary samples, 14 (50%) detected MTB, of which only 1 (7.14%) was RI, 8 (57.14%) were RS, and 5 (35.71%) were RR. On second sample testing, the rates of RR for lymph nodes and tissue samples was 33.33% and 50%, respectively. Eighty-one (69.83%) patients solely received first-line ATT, 7 (6.03%) patients solely received second-line ATT, and 28 (24.14%) patients received both. Of these 28 patients, 20 (71.43%) patients had initially received first-line and were shifted onto second line while 8(28.57%) patients received concurrent first- and second-line ATT.

**Conclusion:**

Repeat pulmonary sample testing in patients with RI results on first sample are less likely to detect MTB and are more likely to be RS, thus, patients may be empirically treated with first-line ATT if a second sample cannot be collected. Repeat extrapulmonary sample testing in patients with RI results on first sample are likely to detect MTB, have actionable results on NAAT, and may have RR results. Thus, collecting a second extrapulmonary sample, particularly lymph nodes and tissues, should be done in those in whom it is feasible.

**Disclosures:**

All Authors: No reported disclosures

